# Irrational Health Behaviour and Existential Drives During Pandemics: Sexual Risk, Psychological Vulnerability, and the Origins of the Samos Syndrome

**DOI:** 10.1192/j.eurpsy.2026.11888

**Published:** 2026-07-31

**Authors:** C. Lazzari

**Affiliations:** Psychiatry, International Centre for Healthcare and Medical Education (ICHME), London, United Kingdom

## Abstract

**Introduction:**

Certain behaviours during pandemics—including intentional exposure to illness despite awareness of risk—reflect an irrational mode of health decision-making. Such actions may emerge from unconscious drives, existential ambivalence, and psychosocial conflict. The phenomenon known as *Samos Syndrome*, inspired by an historical episode documented by Zambaco Pacha and narrated by Ceronetti (1990), illustrates health-risking behaviours driven by symbolic devotion, fatal attraction, or unconscious desire.

**Objectives:**

To define irrational health behaviour in pandemic contexts and analyse how psychological vulnerability and existential factors shape high-risk sexual decision-making. The study examines the psychodynamic foundations of Samos Syndrome and provides a framework for understanding behavioral resistance despite knowledge and awareness of transmission risks.

**Methods:**

A qualitative and conceptual methodology was adopted, drawing on psychoanalytic literature, historical accounts, and clinical interviews. Case studies from HIV/AIDS and COVID-19 crises were analysed alongside theoretical perspectives from Freud, Brenner (1988), and Borlotti (2015). The concept of *capacity assessment* was utilised to evaluate the cognitive and affective conditions underpinning health decisions.

**Results:**

Risky sexual behaviour under existential stress often reflects unconscious conflict between Eros and Thanatos. In high-stimulation settings, individuals may override health knowledge in pursuit of gratification, symbolic meaning, or emotional relief. Samos Syndrome describes such enactments, particularly in populations with depressive or borderline personality features. Observed decisions included disregard for viral latency and self-harm through intimate contact, akin to slow-motion suicide.

**Table 1.** Table 1. long description.

Section	Key Concepts	Analytical Insights	Implications
**Results**	Eros and Thanatos; Samos Syndrome; symbolic gratification; borderline/depressive traits	Sexual risk-taking reflects unconscious conflict and emotional volatility; intimate contact as existential enactment	Highlights health behaviour as psychodynamic expression rather than cognitive failure
**Conclusions**	Impaired capacity; symbolic suffering; psychological vulnerability	Pandemic resistance stems from existential drives and unresolved distress, not ignorance	Calls for psychiatric models that incorporate psychodynamic conflict and affective meaning into prevention design

**Conclusions:**

Irrational health behaviours during pandemics cannot be solely attributed to lack of information. They often derive from impaired reflective capacity, unresolved psychological distress, and symbolic expressions of suffering or desire. Psychiatric interventions must engage with the existential and psychodynamic substrates of decision-making to foster more effective prevention strategies.

**Disclosure of Interest:**

None Declared

## Long descriptions

### Table 1. Long description

The table is organized into four columns: Section, Key Concepts, Analytical Insights, and Implications.

Row 1 (Results):

- Key Concepts: Eros and Thanatos; Samos Syndrome; symbolic gratification; borderline/depressive traits.

- Analytical Insights: Sexual risk-taking reflects unconscious conflict and emotional volatility; intimate contact as existential enactment.

- Implications: Highlights health behaviour as psychodynamic expression rather than cognitive failure.

Row 2 (Conclusions):

- Key Concepts: Impaired capacity; symbolic suffering; psychological vulnerability.

- Analytical Insights: Pandemic resistance stems from existential drives and unresolved distress, not ignorance.

- Implications: Calls for psychiatric models that incorporate psychodynamic conflict and affective meaning into prevention design.

Navigate back to Table 1..

